# Stellate Ganglion Nrf2 Modulates Oxidative Stress and Heart Rate Responses in Mice and Rats with Heart Failure

**DOI:** 10.3390/antiox15091054

**Published:** 2026-08-24

**Authors:** Julia Shanks, Neha Dhyani, Tara L. Rudebush, Lie Gao, Hanjun Wang, Irving H. Zucker

**Affiliations:** 1 Department of Physiology, Faculty of Medical and Health Sciences, Manaaki Manawa the Centre for Heart Research, University of Auckland, Auckland 1010, New Zealand; julia.shanks@auckland.ac.nz; 2 Department Cellular and Integrative Physiology, University of Nebraska Medical Center, Omaha, NE 68198, USA; ndhyani@unmc.edu (N.D.); trudebush@unmc.edu (T.L.R.); 3 Department of Anesthesiology, University of Nebraska Medical Center, Omaha, NE 68198, USA; lgao@unmc.edu (L.G.); hanjunwang@unmc.edu (H.W.)

**Keywords:** ROS, antioxidants, sympathetic nerve activity

## Abstract

Chronic heart failure (CHF) is a growing global health concern characterized, in part, by progressive sympathetic overactivation, which exacerbates this condition. Accumulating evidence identifies oxidative stress as a key driver of sympatho-excitation, mediated by excess reactive oxygen species (ROS) and impaired antioxidant defenses. The redox-sensitive transcription factor nuclear factor erythroid 2–related factor 2 (Nrf2) is a central regulator of antioxidant gene expression, but its role in the peripheral sympathetic nervous system, particularly in the stellate ganglia, remains unclear. We hypothesized that Nrf2 signaling is disrupted in the stellate ganglia in CHF and that modulation of Nrf2 alters ROS levels and sympathetic responses. In rats, six weeks post myocardial infarction (MI), the stellate ganglia exhibited increased ROS levels in tyrosine hydroxylase-positive neurons along with reduced Nrf2 protein and mRNA expression; both changes were inversely associated with ejection fraction (EF). To determine the functional role of Nrf2, lentiviral vectors encoding GFP or GFP-Nrf2 were delivered to the stellate ganglia three weeks after MI. Nrf2 upregulation attenuated heart rate responses to stellate stimulation in sham rats but augmented responses in CHF rats. The increase in plasma norepinephrine levels was reduced following stellate stimulation in CHF rats that overexpressed Nrf2, while β1-adrenergic responsiveness to dobutamine was unchanged. This study demonstrates that CHF is associated with increased oxidative stress and reduced Nrf2 expression in the stellate ganglion. Nrf2 overexpression significantly modulated sympathetic regulation, altering heart rate responses and reducing plasma norepinephrine in CHF rats. These findings support the concept that impaired Nrf2 signaling contributes to ganglionic redox imbalance and dysregulated sympathetic nerve activity in CHF.

## 1. Introduction

Chronic heart failure (CHF) has become epidemic in developed nations, accounting for approximately 6.5 million patients in the US [1,2]. The incidence of CHF continues to grow despite successfully treating acute myocardial infarction (MI) and acute heart failure (HF). The mechanisms by which cardiovascular (CV) function spirals downward in CHF (with either low or preserved ejection fraction (EF)) are multifactorial and involve almost every organ system. Strong evidence points to central sympatho-excitation as contributing to the deterioration of CV function in this disease [3,4,5,6,7]. Previous studies have focused on several mediators (e.g., Angiotensin II (Ang II), nitric oxide (NO), glutamate, norepinephrine (NE)) and central regulation as important contributors to sympatho-excitation in the setting of CHF. The role of oxidative stress in sympatho-excitation in CHF has become prominent in contemporary thinking about this process [8,9,10]. A variety of pro-inflammatory and pro-oxidant factors, including cytokines, NE, NF-ĸB and Ang II are all activated in CHF [11]. Importantly, reactive oxygen species (ROS) are regulated, in part, by the balance between their generation and their scavenging through a variety of antioxidant molecules and pathways. Whether the source of ROS is mitochondrial or cytosolic, a lack of ROS homeostasis results in cellular damage that involves oxidation of DNA, proteins, and lipids.

The redox-sensitive transcription factor nuclear factor (erythroid-derived 2)-like 2 (Nrf2) and its cytosolic tether, Kelch-like ECH-associated protein 1 (Keap1), play important roles in regulating the level of ROS in many tissues [12,13,14]. Data from our laboratory and others show that key antioxidant enzymes are decreased in sympatho-regulatory areas of the brain in animals with CHF, resulting in increased levels of superoxide (O_2_^−^·) [15,16,17]. The binding of Nrf2 to Keap1 and ultimately antioxidant response elements (AREs) is activated by oxidative stressors and electrophiles, including xenobiotics and substances contained in a variety of fruits and cruciferous vegetables (e.g., sulforaphane, curcumin) [18,19]. Activation of this pathway is beneficial and protective in a variety of disease models ranging from CV disease to cancer [20,21]. ROS (primarily H_2_O_2_) [22] have been shown to oxidize critical sulfhydryl groups on many of the 27 cysteine residues of Keap1, causing it to dissociate from Nrf2, phosphorylate, and enter the nucleus to initiate transcription of antioxidant enzymes by binding to AREs [23]. These include NAD(P)H Quinone Oxidoreductase (NQO1), catalase, Heme Oxygenase 1 (HO1), and glutathione peroxidase [24]. Previous work from our laboratory using Nrf2 and Keap1 floxed mice demonstrated the importance of this pathway in the central nervous system and the activation of sympathetic outflow in both normal animals and in those with CHF [25,26,27]. In addition, the balance between Nrf2 and NFkB plays an important role in the regulation of ROS in CHF [28,29]. This competition may serve as a switch to change between pro- and anti-inflammatory signals in neurons, microglia, etc.

In contrast to the large body of work in the central nervous system, few studies have examined the pro- and antioxidant pathways in the peripheral nervous system, especially in sympathetic ganglia in the setting of CHF [30]. Oxidative stress has been associated with cardiac arrhythmias in several disease states [31,32,33]. Several studies have now implicated oxidative stress in the stellate ganglia (SG) as contributing to arrhythmias and enhanced sympathetic outflow in animals and humans [34,35,36].

Therefore, the goal of the current study was to evaluate oxidative stress and Nrf2 in the stellate ganglia of normal animals and those with CHF. We hypothesized that Nrf2 expression in the stellate ganglion is decreased in CHF. In addition, we sought to determine whether upregulation of stellate ganglion Nrf2 altered ROS and the response to stellate ganglion stimulation in rodents.

## 2. Methods

All procedures were approved by the Institutional Animal Care and Use Committee of the University of Nebraska Medical Center (IACUC protocol #18-174-02-FC; approved 20 February 2019) and performed in accordance with the National Institutes of Health’s Guide for Use and Care of Laboratory Animals.

Mice and rats were used in these studies to show the ubiquity of the changes in Nrf2 in the stellate ganglion. Rats were used for viral transfection studies and stellate stimulation studies due to their size and ease of injection and stimulation.

Wild-type male and female C5BL6J mice were purchased from Jackson Laboratories (Bar Harbor, ME, USA). Mice started the study at 6–8 weeks of age, weighing approximately 18–23 g for females (n = 14) and 26–30 g for males (n = 41). Adult male Sprague–Dawley rats aged ~3 mo and weighing 180–200 g (n = 42) were purchased from Charles River, Inc. (Wilmington, MA, USA). Animals were group-housed and subjected to a 12 h light–dark cycle. Mouse (Teklad 7012) or rat (Teklad 8653) laboratory chow and water were available ad libitum.

### 2.1. Model of Chronic Heart Failure

Heart failure in mice and rats was produced by permanent coronary artery ligation as previously described [25,37]. Briefly, animals were anesthetized with isoflurane (2–3%) and mechanically ventilated (mice: 120 breaths/min, rats: 60 breaths/min). A left thoracotomy was performed between the 4th and 5th rib, the pericardium was opened, the heart was visualized, and the left anterior descending coronary artery was ligated (mice: 8-O silk suture, rats: 6-O silk suture). The ribs and skin layers were closed separately with a suture (4-O monofilament). Sham-operated animals underwent the same procedure but did not undergo coronary artery ligation. All mice and rats survived the sham surgery. Approximately 70% of mice and 80% of rats survived the coronary artery ligation surgery. All animals were given subcutaneous saline (0.2 mL for mice and 1 mL for rats) and buprenorphine (1 mg/kg sc) post-surgery and recovered in a cage on a heating pad and with supplemental oxygen.

### 2.2. Echocardiography

Cardiac function in all experimental animals was measured under isoflurane (1–2%) at three and six weeks after MI by echocardiography (VEVO 3100, Fujifilm VisualSonics, Inc., Toronto, ON, Canada). Cardiac parameters measured included ejection fraction (EF), fractional shortening (FS), cardiac output and cardiac volumes. An EF cut-off of 45% was used to define heart failure in mice and rats. All terminal experiments in rats and mice were performed 6 weeks after MI or sham surgery.

### 2.3. Rat Viral Gene Transfer

A custom lentiviral vector for targeted Nrf2 overexpression within sympathetic neurons (HIV-PRSx8-eGFP-Nrf2 VSVG 2.2 × 10^8^ TU/mL; University of Iowa Viral Vector Core, Iowa City, IA, USA) was used. A second lentivirus, which expressed GFP but not Nrf2 (HIV PRSx8-eGFP VSVG 1.6 × 10^8^ TU/mL; University of Iowa Viral Vector Core), was used to control for the effect of mechanical injection of the stellate and viral infection. Three weeks post-MI surgery, heart failure was confirmed by echocardiography, and the rats underwent a second surgery in which a lentiviral vector was injected into the right stellate ganglion. HF and sham rats were anesthetized with isoflurane (2–3%) and placed in the prone position. Using a surgical microscope, a small surgical incision was made between the first and second rib, close to the spinal cord where the right stellate ganglion was exposed. A 10 µL Hamilton syringe was used to inject 3–5 µL of viral vector (with GFP or GFP and Nrf2) into the stellate ganglia. Injection of the ganglia was confirmed with visual and physical puncture of the connective tissue encasing the stellate, with care taken not to break the endothoracic fascia. The skin incision was sutured closed, and the animals recovered in a cage, partially on a heating pad, before being returned to their home cage.

Sham (n = 19) and MI (n = 23) rats were assigned to GFP or GFP-Nrf2 at random. This resulted in four groups of rats: Sham-GFP (n = 10), Sham-Nrf2 (n = 9), MI-GFP (n = 11) and MI-Nrf2 (n = 12). The rats were then housed for a further three weeks before receiving a 6-week post-MI echo and entering terminal experiments.

### 2.4. Immunostaining

The stellate ganglia from a subset of mice and rats were used for immunostaining of redox markers (mice) and confirmation of viral gene transfer (rats). Briefly, 6 weeks post-MI, animals were euthanized with an overdose of pentobarbital sodium (150 mg/kg) and perfused with cold PBS through the apex of the left ventricle for 20 min before switching to cold 4% PFA for 20 min. The stellate ganglia were removed and post-fixed in 4% PFA overnight. The stellate ganglia were cryoprotected in a graded sucrose series (up to 30%) and embedded in optimal cutting temperature (OCT) compound. Frozen sections were cut at 14 µm on a cryostat and mounted on glass slides.

To facilitate antibody penetration, sections were permeabilized with 0.2% Triton X-100 in PBS for 20 min at room temperature, followed by a 1 h incubation in a blocking solution consisting of 5% fetal bovine serum (FBS) in PBS. The sections were then incubated overnight at 4 °C with primary antibodies (8-hydroxy-2′-deoxyguanosine (8-OHdG; 1:50, mouse monoclonal, Santa Cruz Biotechnology, sc-393871); 4-Hydroxynonenal (4-HNE; 1:100, mouse monoclonal, Abcam (Cambridge, UK), ab48506); nuclear factor erythroid 2–related factor 2 (Nrf2; 1:100, rabbit polyclonal, Abcam, ab31163); 3-Nitrotyrosine (3-NT; 1:100, mouse monoclonal, Abcam, ab110282); tyrosine hydroxylase (TH; 1:100, chicken polyclonal, Abcam, ab76412)) diluted in blocking buffer. Following primary incubation, sections were washed three times in PBS (5 min per wash) and incubated with the following fluorophore-conjugated secondary antibodies (Goat anti-chicken Alexa Fluor 568 (for TH); Goat anti-mouse Alexa Fluor 405 (for 8-OHdG, 4-HNE, or 3-NT); Chicken anti-rabbit Alexa Fluor 594 (for Nrf2)) in 1% FBS for 1 h at 37 °C. Slides were washed 3 times in PBS, and coverslips were mounted using Vectashield Antifade Mounting Media (Vector Laboratories, Newark, CA, USA). The slides were stored at 4 °C and protected from light until imaging. Negative controls, performed by omitting the primary antibodies, were used to confirm the staining’s specificity and the absence of nonspecific secondary binding. Images were acquired on a laser confocal microscope (Zeiss 710, Carl Zeiss AG, Jena, Germany) at appropriate wavelengths.

### 2.5. Western Blotting

A subset of mice were euthanized by CO_2_ inhalation. Bilateral stellate ganglia were rapidly dissected and finely minced using sterile surgical scissors. The tissue fragments were transferred into pre-chilled microcentrifuge tubes containing radioimmunoprecipitation assay (RIPA) buffer (50 mM Tris-HCl, 195 mM NaCl, 2 mM EDTA, 1% NP-40, 0.1% SDS) supplemented with 1% protease inhibitor cocktail (Abcam, Cambridge, UK; ab65621). The samples were homogenized, centrifuged, and stored at −80 °C until further use. Protein concentrations were determined using a Pierce protein assay kit (Rockford, IL, USA).

Equal amounts of protein (15 μg in 15 μL per sample) were separated on 10% SDS-PAGE gels alongside Invitrogen MagicMark™ Western Standard (Invitrogen, Waltham, MA, USA) and Bio-Rad Precision Plus Protein Dual Color Standards (Bio-Rad, Hercules, CA, USA) loaded in separate lanes. Electrophoresis was performed using a Mini Gel Tank and Blot Module Set (NW2000, Invitrogen) at 70 V for approximately 15 min, followed by 120 V until adequate separation was achieved. Proteins were then transferred onto nitrocellulose membranes using the iBlot2 system (IB21001, Thermo Fisher Scientific, Waltham, MA, USA) with preset program P0 (20 V for 1 min, 23 V for 4 min, and 25 V for 2 min).

Membranes were briefly rinsed with Milli-Q water and blocked for 30 min at room temperature in 5% nonfat milk prepared in 1× PBST, followed by two washes (5 min each) in 1× PBST. Membranes were then incubated with primary antibodies against nuclear factor erythroid 2–related factor 2 (Nrf2; ab62352, Abcam, Cambridge, UK), NAD(P)H Quinone Dehydrogenase 1 (NQO1; ab80588, Abcam, Cambridge, UK), and GAPDH (1:1000; sc-32233, Santa Cruz Biotechnology, Dallas, TX, USA). The following day, membranes were washed three times for 5 min each in 1× PBST and subsequently incubated with the appropriate secondary antibodies.

Protein bands were visualized using an enhanced chemiluminescence reagent (Pierce Chemical, Rockford, IL, USA) and imaged with the G:Box system using Genesys software (v1.5; SYNGENE, Cambridge, UK). Densitometric analysis was performed, and Nrf2 and NQO1 expression levels were normalized to GAPDH.

### 2.6. Plasma NE Measurement

Blood samples collected after the stellate stimulation protocol were centrifuged, and the plasma was removed. Venous blood samples were collected via a catheter in the left jugular vein. Blood was slowly withdrawn and then transferred into a 10 mL K2 EDTA Plus Blood Collection Tube (BD Vacutainer, REF 366643). The samples were frozen at −80 °C until use. The samples were centrifuged at 1000× *g* for 10 min at 4 °C to yield approximately 5 mL of plasma. From this, 300 μL was used to measure norepinephrine (NE) concentrations using a Norepinephrine Enzyme Immunoassay kit (Labor Diagnostika Nord KG, Nordhorn, Germany) according to the manufacturer’s instructions. All samples were measured in duplicate.

### 2.7. Terminal Experiment Protocols-Rats

Six weeks post-MI or sham surgery and 3 weeks post-viral transfection, all rats entered a terminal experiment protocol. The rats were anesthetized with isoflurane (1–3%). A Millar catheter (Model #SPR-524 and Size 3.5 F, Millar Instruments, Houston, TX, USA) was advanced through the carotid artery into the left ventricle (LV) to determine LV pressure and LV dp/dt max and dp/dt min. A three-lead ECG was placed across the left upper limb, right lower thorax and abdomen. A venous line was placed in the left jugular vein for fluid and drug infusion and blood sampling. The right side of the chest was opened, and the intercostal space between the second-third rib was opened to expose the stellate ganglia. Using a dissecting microscope, a stainless-steel electrode was pressed against the ganglia. The electrode was connected to a Grass stimulator. Out of 27 rats, 6 died (3× Sham-GFP, 1× Sham-Nrf2, 1× MI-GFP and 1× MI-Nrf2) during the instrumentation phase and did not complete the protocol; all but one were then used for molecular analysis.

### 2.8. Stellate Stimulation

In anesthetized rats, following a 20–30 min baseline stabilization period, the rats underwent right stellate ganglia stimulation. The stellate ganglion was electrically stimulated at 0.5 Hz, 1 Hz, 3 Hz, 5 Hz, and 7 Hz in a randomized order. Each stimulus was delivered at 5 V, with a 0.5 ms pulse width for 30 s, with 5 min between stimulations. At the baseline prior to and at the end of the stellate stimulation protocol, venous blood samples were taken for plasma NE measurements. At the end of the experiment, the animals were euthanized with pentobarbital sodium (150 mg/kg). The hearts and lungs were removed, and the ratio of the infarct area to the whole LV minus the septum was measured.

### 2.9. Dobutamine Injections

To determine ß-1 receptor responsiveness following stellate stimulation, the rats were administered an intravenous dose–response of dobutamine (Sigma-Aldrich (St. Louis, MO, USA), cat #D0676; 0.01 µg/kg, 0.05 µg/kg, 0.1 µg/kg, 0.5 µg/kg, 1 µg/kg, 5 µg/kg). Each dose was flushed with 100 µL saline.

### 2.10. Metoprolol

Five minutes after the last dose of dobutamine, a single 5 Hz stellate stimulation was given to confirm that the response was comparable to the previous stimulations and that the preparation remained responsive. A single dose of metoprolol (3 mg/kg; metoprolol tartrate, Sigma Aldrich) was given intravenously, and the stellate stimulation protocol was repeated.

### 2.11. Statistics

All data in the graphs and tables are presented as mean ± SD. Comparison of single timepoint data, changes in resting hemodynamics, Nrf2 protein, mRNA and plasma NE were made using unpaired Student’s *t*-test. Scatter plots were fitted with simple linear regression. Comparison of between-group differences in stellate ganglia stimulation were made using a repeated-measure two-way ANOVA. GraphPad Prism 10.0 (GraphPad Software, San Diego, CA, USA) was used for graphing and statistical analysis. *p* < 0.05 was considered significant.

## 3. Results

**Figure 1:**
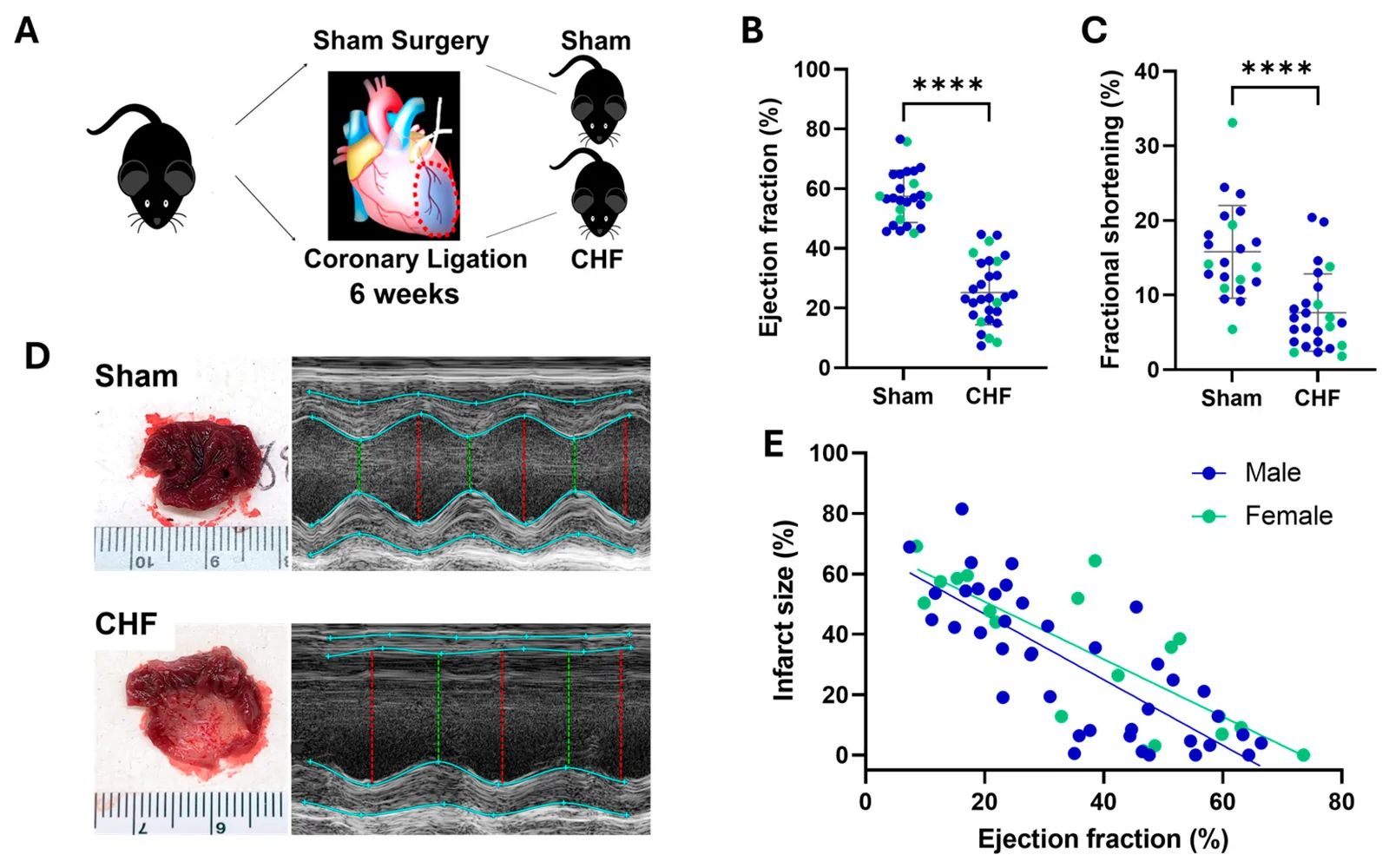
(**A**) Schematic representation of mouse myocardial infarction (MI) protocol. Six weeks post-MI (n = 29) or sham (n = 26) surgery, mice with chronic heart failure (CHF) had reduced ejection fraction (**B**) and fractional shortening (**C**). (**D**) Representative images for infarction size and echocardiography, left ventricle short-axis m-mode for sham and CHF mice. Red line denotes end diastolic diameter; green line denotes end systolic diameter. (**E**) Infarction size correlated with ejection fraction; male, R^2^ = 0.62, *p* < 0.0001. female, R^2^ = 0.64, *p* < 0.0001. Unpaired *t*-test **** *p* > 0.001.

To characterize the CHF model and determine whether cardiac dysfunction was associated with alterations in stellate ganglion redox signaling, C57B6 mice were subjected to coronary artery ligation or sham surgery as shown in Figure 1. There were profound decreases in EF and FS in both male and female mice 6 weeks after coronary artery ligation surgery. Figure 1D provides examples of infarct size and M-mode echocardiography of sham and CHF mice. Sham animals exhibited no signs of cardiac injury, while the mean infarct size in CHF mice (male and female combined) was 41.7 ± 4.1 percent of the left ventricle. Heart weight/body weight ratio was significantly increased in MI mice (145.8 ± 31.9 vs. 202.3 ± 83.3; *p* < 0.05) (Table 1). All mouse hemodynamic parameters are shown in Table 1, and sex-specific data are shown in Table 2. In addition to reduced EF and FS, there was a significant decrease in the left ventricular dp/dt max and min. As is typical of this model, infarct size was inversely (Figure 1E) correlated with EF in both male and female mice.

**Figure 2:**
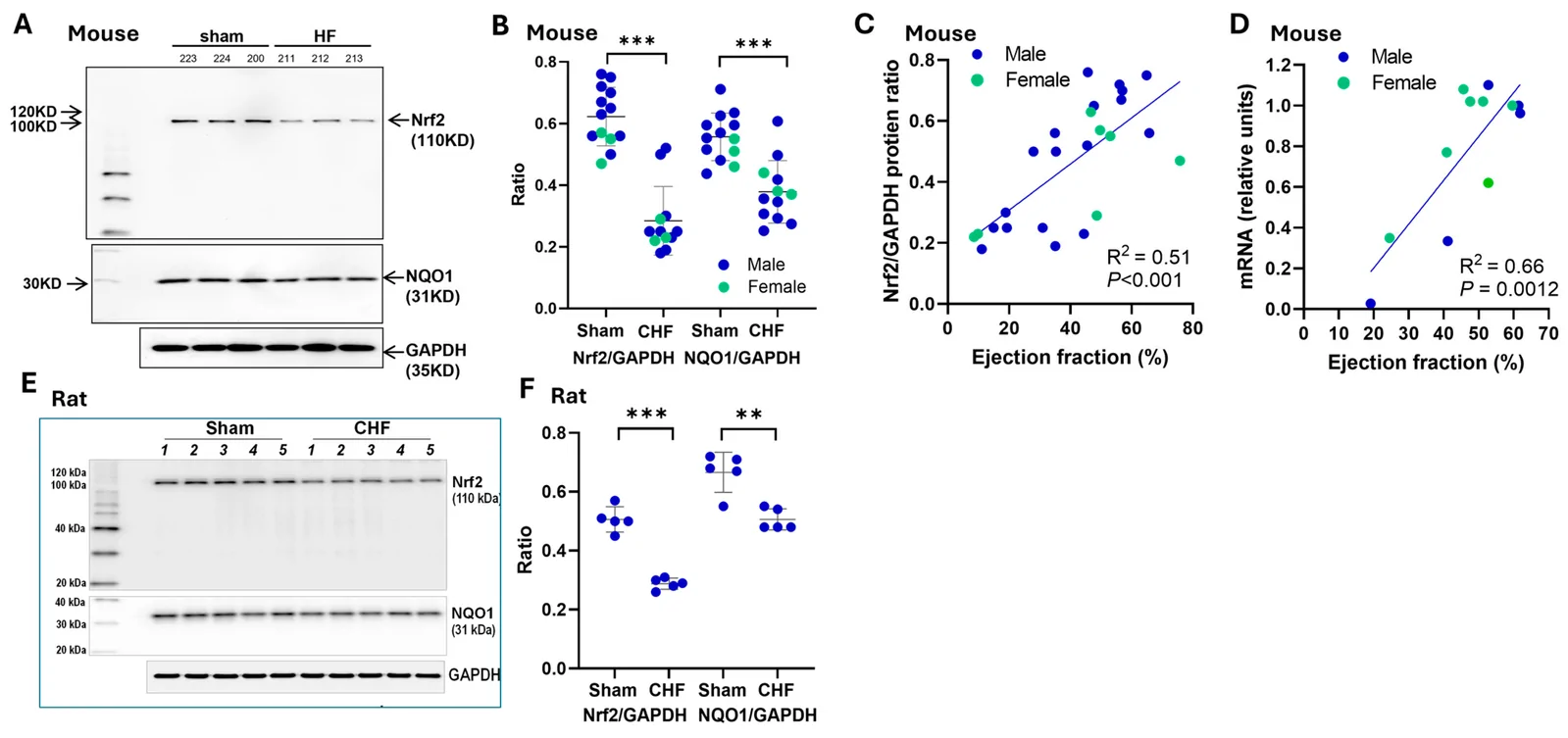
(**A**) Representative Western blot for Nrf2 and NQO1 from sham and CHF mouse stellate ganglia (n = 12–13, all groups). (**B**) Group data presenting protein expression in relation to GAPDH in mice (n = 25). (**C**) Relationship between stellate ganglia Nrf2/GAPDH protein expression and ejection fraction in mice. (**D**) Relationship between stellate ganglia Nrf2 mRNA and ejection fraction in mice (n = 12, paired left and right stellate ganglia). (**E**) Representative Western blot for Nrf2 and NQO1 from sham and CHF rat stellate ganglia (n = 5, all groups). (**F**) Group data presenting protein expression in relation to GAPDH in rats. ** is *p* < 0.005, *** *p* < 0.001.

To determine whether CHF altered antioxidant signaling within the stellate ganglia, Nrf2 expression was evaluated. CHF significantly reduced Nrf2 protein and mRNA expression in mouse stellate ganglia accompanied by a decrease in the downstream antioxidant protein NQO1 (Figure 2). There were no sex differences observed. A significant correlation existed between the level of Nrf2 protein and mRNA and EF (Figure 2C,D). As in mice, CHF rats showed significantly lower stellate Nrf2 and NQO1 protein expression compared with sham animals (Figure 2E,F). Nrf2 mRNA was not measured in rat stellate ganglia. No sex-dependent differences were detected.

**Figure 3:**
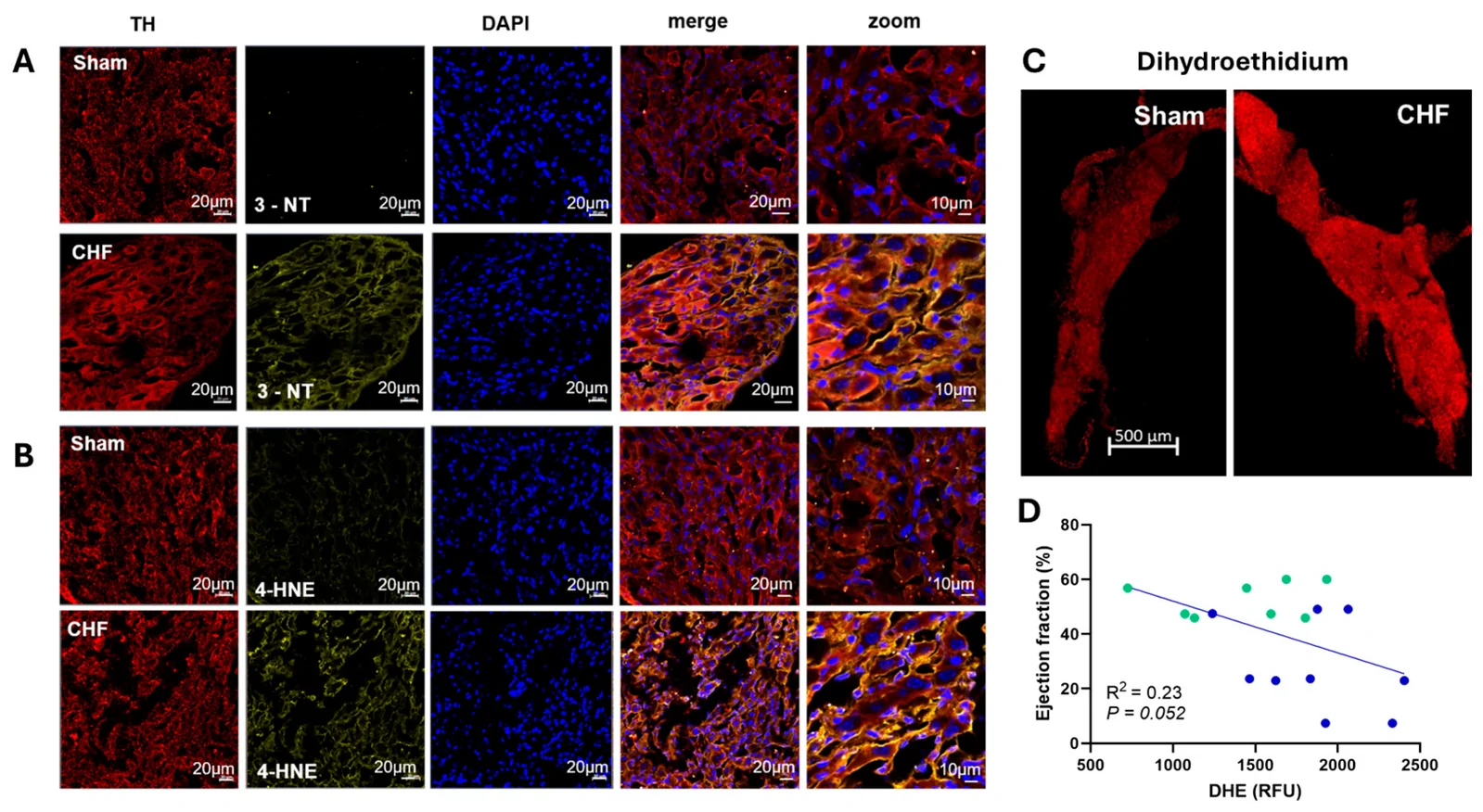
Representative images from the stellate ganglia of sham and CHF mice. PFA fixed and sliced sections of stellate ganglia from sham and CHF mice labeled with TH-tyrosine hydroxylase. (**A**) 3-Nitrotyrosine (3-NT), (**B**) 4-hydroxynonenal (4-HNE). (**C**) Fresh, whole mount imaging of stellate ganglia incubated with dihydroethidium (DHE). (**D**) DHE fluorescent intensity compared to ejection fraction in mouse stellates, green dots—sham mice, blue dots—myocardial infarction mice.

To determine if reduced Nrf2 signaling was associated with increased oxidative stress in the stellate ganglia of mice with CHF, we examined immunofluorescence images in tyrosine hydroxylase (TH)-positive neurons using three different ROS markers. Figure 3A,B show examples of increases in 3-Nitrotyrosine (3-NT) and 4-hydroxynonenal (4-HNE), while Figure 3C shows increases in dihydroethidium (DHE) staining in a sham and HF mouse. Importantly, an inverse relationship was observed between DHE expression and EF (Figure 3D).

Because our subsequent studies required selective gene delivery into the stellate ganglia and functional assessment during stellate stimulation, these experiments were performed in Sprague–Dawley rats in which these procedures are technically more feasible. Before initiating these intervention studies, we confirmed that the rat CHF model exhibited changes similar to those observed in mice (see Table 3).

To determine if upregulating Nrf2 expression in the stellate ganglia could modify sympathetic function, rats with and without CHF received bilateral stellate injections of either a Nrf2-expressing lentiviral vector or a control GFP vector.

**Figure 4:**
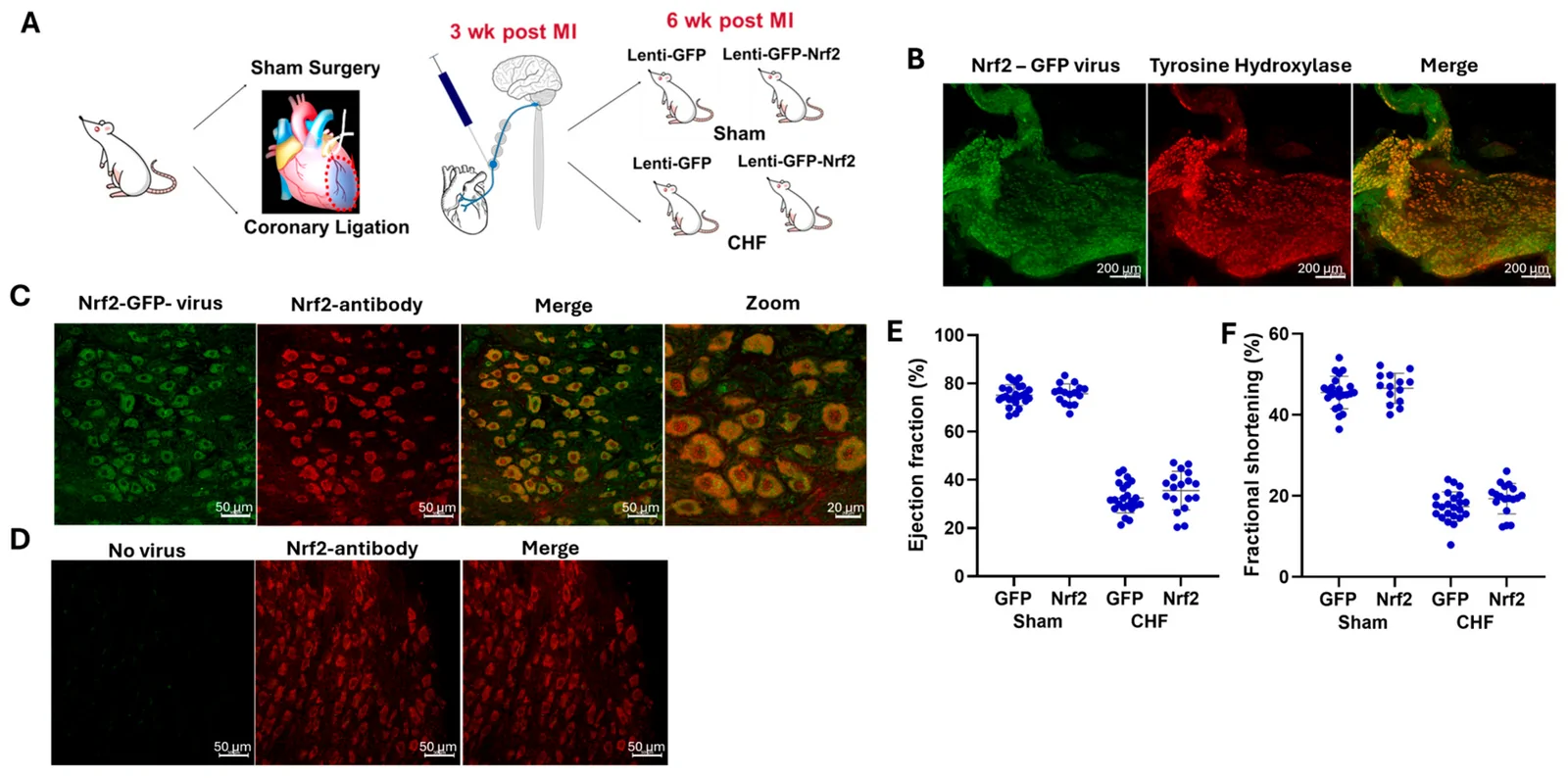
(**A**) Schematic of the MI rat, Nrf2 upregulation by viral transfection protocol. (**B**) Representative image showing Nrf2-GFP viral transfection in tyrosine hydroxylase-positive cells from the rat stellate ganglion. (**C**) Images from slices of rats stellate ganglion transfected with lenti-Nrf2-GFP and Nrf2 antibody. (**D**) Stellate ganglia without viral transfection and Nrf2 antibody labeling. Rats with MI show reduced ejection fraction (**E**) and fractional shortening (**F**) compared to sham. Stellate ganglia upregulation of Nrf2 had no effect on any echocardiography measures, including ejection fraction and fractional shortening 3 weeks after transfection.

Figure 4 shows representative images of successful viral overexpression of Nrf2 in TH-expressing neurons in the stellate ganglion of rats. In both whole stellate and in sections of stellates (Figure 4B–D), Nrf2 viral transfection increased Nrf2 protein expression despite no alterations in EF or FS (Figure 4E,F).

**Figure 5:**
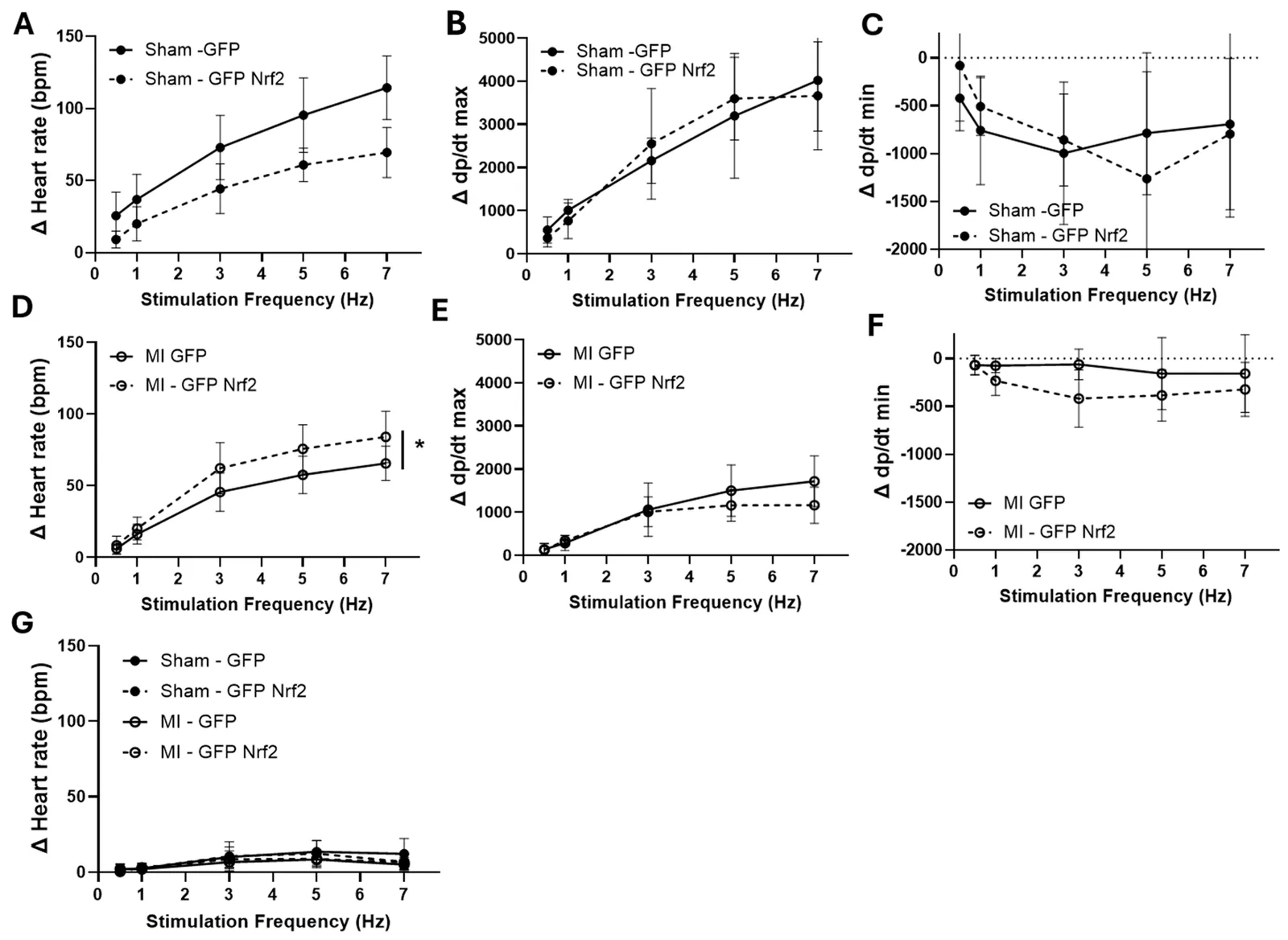
Cardiac changes to right stellate ganglia stimulation. (**A**–**C**) Sham rats (Sham-GFP and Sham-Nrf2, n = 5, both groups). (**A**) changes in heart rate; (**B**) changes in dp/dt max; (**C**) changes in dp/dt min. (**D**–**F**) MI-GFP and MI-GFP Nrf2 rats. (**D**) changes in heart rate (MI-GFP and MI-Nrf2 both n = 6 both groups); (**E**) changes in dp/dt max; (**F**) changes in dp/dt min; (**G**) changes in heart rate after metoprolol treatment, (MI-GFP, n = 5; MI-Nrf2, n = 6), * *p* < 0.05, two-way ANOVA repeated measures—interaction and group.

**Figure 6:**
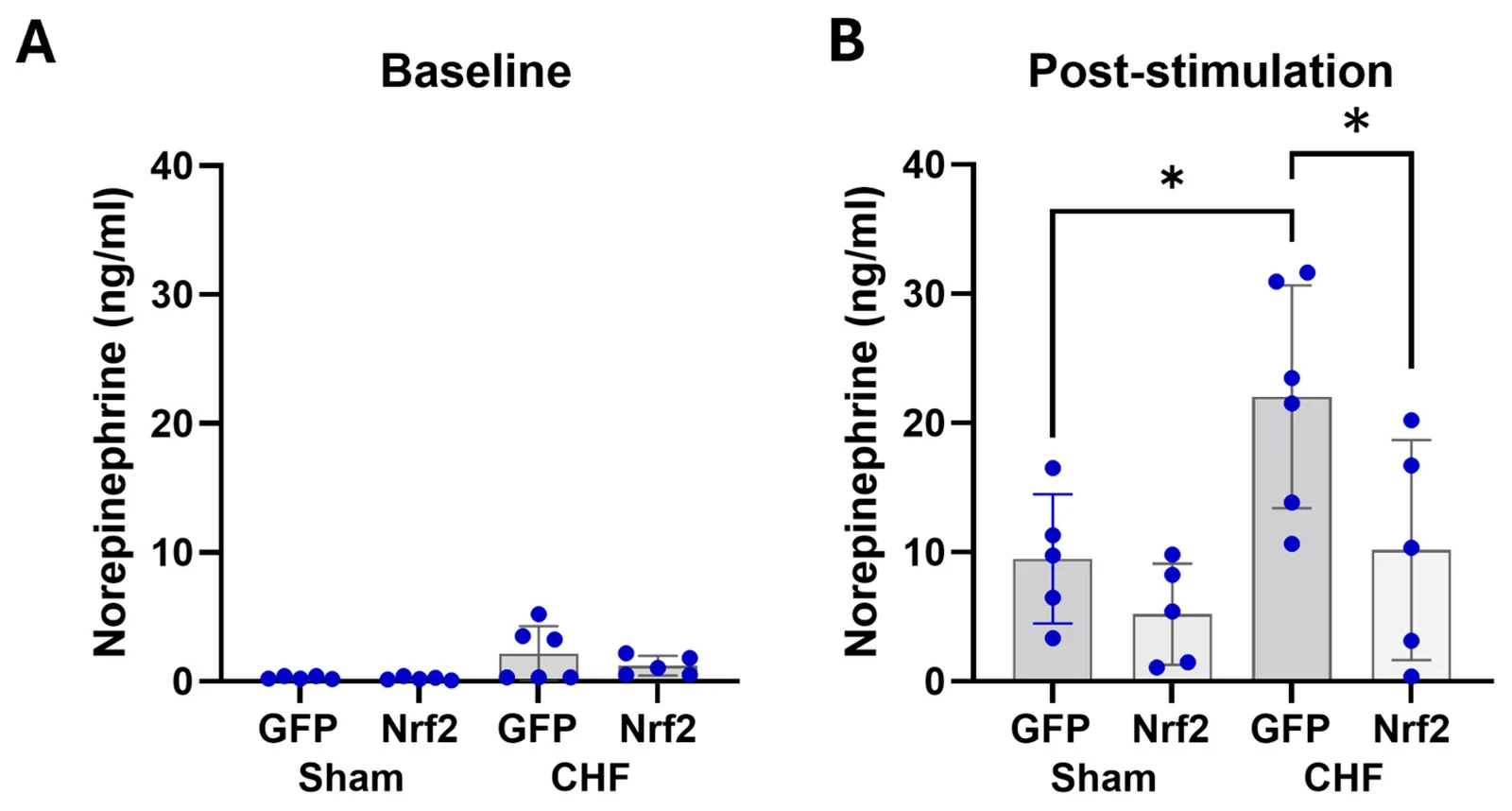
Plasma NE before (**A**) and after (**B**) stellate stimulation in GFP and Nrf2 virally transfected rats. (n = 5–6, all groups) * *p* < 0.05.

We next examined whether increasing stellate Nrf2 expression altered cardiac responses to stellate stimulation. In sham rats, the change in HR was significantly blunted when Nrf2 was upregulated (Figure 5A). On the other hand, in CHF rats, the reverse was observed. That is, Nrf2 upregulation augmented the HR responses to stellate stimulation (Figure 5D). All changes in HR were abolished by treatment with the ß1 antagonist, metoprolol (3 mg/kg; Figure 5G). Viral transfection did not alter the dp/dt max or min responses to stellate stimulation between groups. Additional evidence indicating a reduction in sympatho-excitation in rats with stellate overexpression of Nrf2 is shown in Figure 6 where plasma NE levels were measured before and after stellate stimulation in sham and CHF rats. Nrf2 overexpression had no effect on baseline or stimulated NE levels in either group. However, CHF animals exhibited significantly higher plasma NE following stimulation compared to sham animals (Figure 6B). Nrf2 overexpression in the CHF group significantly attenuated this response.

**Figure 7:**
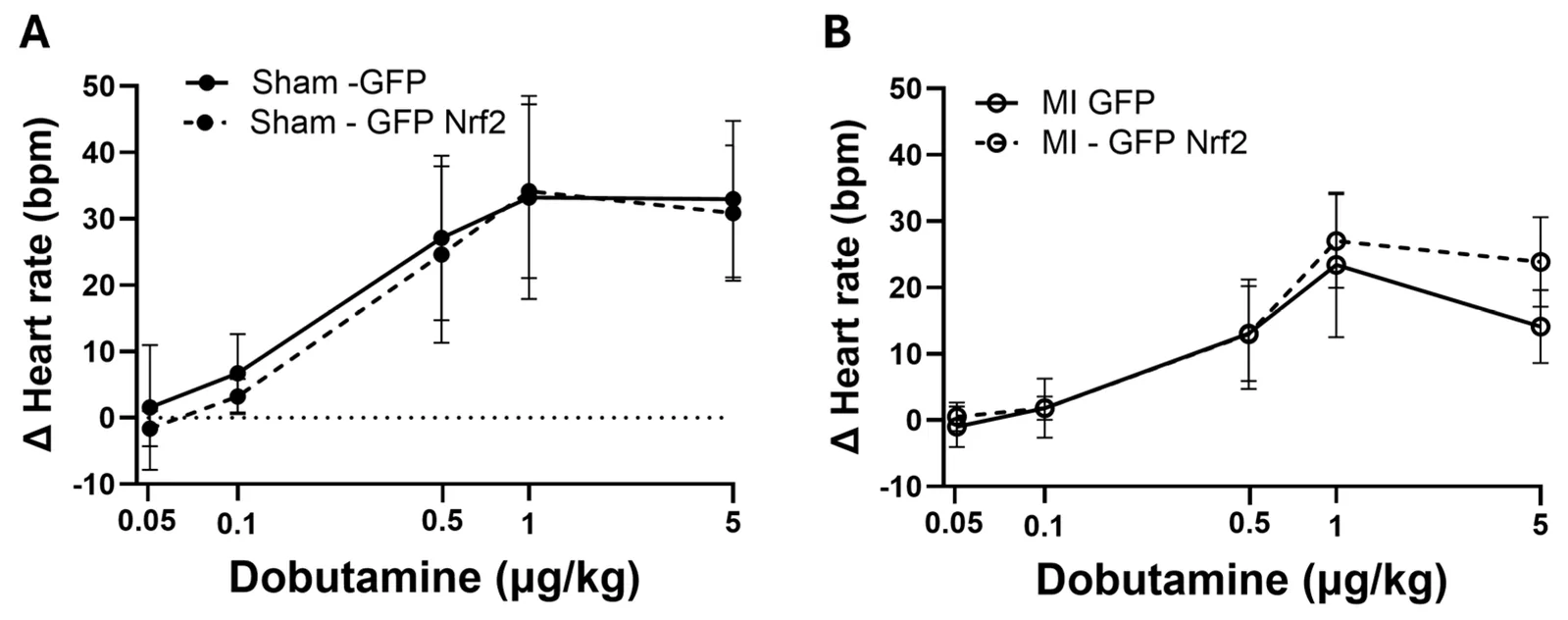
Hemodynamic changes to intravenous dobutamine in anesthetized rats. (**A**) Sham; (**B**) rats with MI, (n = 5, all groups). two-way ANOVA-group: Sham-GFP–MI GFP.

To rule out alterations in ß1-adrenergic signaling as a confounder to the HR changes in response to stellate stimulation before and after Nrf2 upregulation, we evaluated the change in HR in response to varying doses of dobutamine (Figure 7). There were no differences in the HR response in either sham or MI rats when transfected with either GFP or Nrf2.

## 4. Discussion

The present study evaluated the potential role of Nrf2 in the stellate ganglia of mice and rats with post-MI-induced CHF. Based on our previous studies in the central nervous system [25,26,27], we hypothesized that a decrease in stellate ganglion Nrf2 contributes to a reduction in antioxidant enzyme expression and an increase in oxidative stress, thus potentially leading to a sensitization of stellate neurotransmission. Our data show an increase in oxidative stress in the whole stellate and in TH-expressing neurons in the CHF state. Three discrete and targeted ROS markers were used to show elevated oxidative stress in post-MI animals. The ubiquity of these changes suggests intense global cellular oxidative stress in stellates in the post-MI state. Importantly, there was a tight correlation between stellate Nrf2 expression and EF, suggesting that the severity of CHF is coupled to ganglionic oxidative stress and perhaps inflammation. It is of interest that viral upregulation of ganglionic Nrf2 did not alter cardiac function per se in either sham or CHF animals. This is likely due to the fact that sham animals already had low levels of oxidative stress and CHF animals had large myocardial infarctions, which were incapable of exhibiting enhanced cardiac function in response to sympathetic activation.

An unexpected finding in the current study was the differential HR responses to the right stellate stimulation in sham and CHF animals with and without viral upregulation of stellate Nrf2 (Figure 5). Viral upregulation of Nrf2 reduced the HR response in sham animals (compared to GFP) but enhanced the response in CHF rats. This apparent contradiction may have several explanations, but the most plausible is that endogenously higher levels of oxidative stress in CHF animals evoke a chronically enhanced cardiac sympathetic outflow, thus downregulating ß1-adrenergic signaling. Upon Nrf2 upregulation, chronic ROS is decreased along with sympathetic outflow, thus upregulating the ß1-adrenergic function. In CHF animals, the enhanced HR response to stellate stimulation following Nrf2 upregulation may be mediated by reduced ROS and reduced sympathetic outflow, thus upregulating ß1 signaling. Therefore, we examined the HR responses to the ß1 agonist, dobutamine. Unfortunately, although there was a trend toward improved dobutamine-induced tachycardic responses in the CHF + Nrf2 overexpression group, no significant differences were observed in the dose–response to intravenous dobutamine between the viral GFP- and Nrf2-treated groups. It should be noted that the responses to dobutamine were far less than those evoked by stellate stimulation (Figure 7). Therefore, the explanation of this paradox remains unclear. Although Nrf2 overexpression in the stellate did not alter cardiac function per se, further studies will have to be done to determine the role of Nrf2 and ROS in arrhythmogenesis per se. It is also possible that upregulation of Nrf2 evokes differential responses to the levels of oxidative stress in sham or MI animals depending on the balance between oxidative and reductive stress known to occur if antioxidant pathways are upregulated to the level where ion channel function and cardiac function may be impacted [38,39].

It is well known that cardiac sympathetic activation results in an increase in plasma NE which is augmented in the CHF state. This NE spillover is mediated by both a decrease in NE uptake and enhanced post-ganglionic release [7]. While stellate stimulation evoked an increase in plasma NE in both sham and CHF groups, there was a significantly greater increase in CHF animals compared to sham. This is consistent with clinical observations [40,41,42]. Importantly, this increase was attenuated by Nrf2 upregulation in CHF rats but not in sham rats (Figure 6). It should be made clear, however, that the assumption that upregulation of Nrf2 translates to lower ROS was not provided in this study.

The findings of the present study are consistent with both preclinical and clinical studies which show that enhanced stellate ganglion activity and remodeling occur in the post-MI state even following short-term ischemia and contribute to arrhythmogenesis [43,44,45,46]. Ajijola and co-workers recently provided evidence for enhanced oxidative stress in the stellates removed from humans with uncontrolled arrhythmia and electrical storm [35]. This study also showed evidence for microglial activation in the stellates of these patients. There have been no studies in which the role of the transcription factor, Nrf2, has been examined in the stellates of animals or humans with CHF. In a recent study from our laboratory [30] we showed marked inflammation and macrophage activation in thoracic dorsal root ganglia in CHF rats consistent with a decrease in Nrf2 and an increase in NFkB and pro-inflammatory cytokine expression. Furthermore, this study showed that sensitization of afferents in DRGs was correlated with a decrease in potassium channel protein expression and altered depolarization. The role of macrophage activation in modulating neuronal excitability, increasing sympathetic outflow, and arrhythmia susceptibility is becoming increasingly well-established [35,47]. In the current study Nrf2 overexpression was driven by a PRSx8 promoter, therefore only allowing overexpression of Nrf2 in cells expressing Phox2a/b, specific to catecholaminergic neurons and, as far as we are aware, absent from glial cells. This highlights the potential that while neural inflammation/oxidative stress may drive sympatho-excitation in CHF, increasing Nrf2 expression in stellate ganglion catecholaminergic neurons can reduce this phenotype without altering microglia cells [47].

The above discussion raises the question of cause and effect. Based on previous studies from this laboratory we believe that CHF and hypertension mediate the reduction in Nrf2 in several tissues including the stellate ganglion. Tian et al. [48] showed that post-MI CHF in rats was associated with an increase in specific circulating microRNAs that target the translation of Nrf2 in the brain. Furthermore, Gao et al. [26] clearly showed that inhibiting Nrf2 gene expression in the rostral ventrolateral medulla in conscious mice evoked sympatho-excitation resulting in hypertension.

It is well known that oxidative stress in the form of superoxide anion or H_2_O_2_ is capable of modifying ion channel proteins leading to enhanced current—voltage relationships [49]. It is reasonable to assume that the increases in oxidative stress observed in the stellates in the current study are accompanied by ion channel modifications and enhanced excitability. Strategies targeting the upregulation of Nrf2 and antioxidant enzyme expression are likely to mitigate arrhythmogenesis.

There are several limitations to the current study that may necessitate further experimentation. We did not actually evaluate arrhythmia potential in this study. It is difficult to induce a significant number of arrhythmias in rodents unless sympathetic nerve activity is markedly augmented in the chronic state. While there is likely to be low-level sympatho-excitation at rest, one would have to impose a stress to evoke arrhythmias via enhanced stellate neurotransmission. Furthermore, we did not record post-ganglionic action potentials or membrane currents to determine the effects of Nrf2 modulation on depolarization dynamics, although a previous study from our laboratory showed a reduction in sodium channel activity in dorsal root ganglia of CHF rats that was reversed by the SOD mimetic, Tempol [50]. Second, no sex differences were observed, although fewer females were included, and the study is not adequately powered for a full sex-stratified analysis. Finally, we did not measure the ß1-adrenergic receptor protein or signaling in this study. It is therefore difficult to determine if differences observed were mediated by up or down regulation of adrenergic signaling at the myocyte level even though the dobutamine studies would suggest not.

While our Western blot analyses focused on Nrf2 and NQO1 proteins, there is ample evidence that Nrf2 impacts gene and protein expression for a variety of antioxidant proteins including SOD, GSTA2, HO-1 and catalase in many tissues [51]. NQO1 is one of the more robust targets and significantly impacts mitochondrial metabolism; thus this was chosen to demonstrate one downstream target of Nrf2. While DHE staining was selected as a measure of superoxide production in the stellate ganglia, it is acknowledged that DHE may also react to other oxidants.

Finally, the HR data presented in Figure 5 is only based on the right stellate stimulation. The left stellate innervates much of the left ventricular and left atrial myocardium. Thus, additional experiments are required to evaluate differences between the right and left.

In conclusion, the results of the current study provide the first evidence, to our knowledge, that stellate ganglion ROS is increased in the CHF state and correlates with the expression of the ubiquitous transcription factor, Nrf2. While there seems to be a disconnect between the HR changes and the plasma NE when Nrf2 was overexpressed in the stellate, future experiments should focus on the cellular mechanisms for this observation. These data may have translational potential in the pharmacological targeting of Nrf2 or Keap1 to reduce stellate ganglion excitability in cardiovascular disease associated with arrhythmogenesis.

## Figures and Tables

**Table 1:** Hemodynamics and echocardiography parameters of sham and MI mice.

		Sham	MI
	Number (Female)	26 (7)	29 (7)
	Body weight (g)	25.4 ± 3.8	26.2 ± 3.6
	Heart weight (mg)	145.8 ± 31.9	202.3 ± 83.3 *
	Lung weight (mg)	171.2 ± 57.1	188.7 ± 60.0
	HW/BW ratio	5.8 ± 1.1	7.8 ± 3.2 *
	LW/BW ratio	6.6 ± 1.8	7.2 ± 2.0
ECHO	Infarct (%)	-	41.7 ± 21.9
	Ejection fraction (%)	57.4 ± 8.7	25.2 ± 10.8 *
	Fractional shortening (%)	15.8 ± 6.2	7.7 ± 5.2 *
	Cardiac output (mL/min)	13.6 ± 4.0	11.8 ± 4.4
	Heart rate (bpm)	459 ± 51.7	470.9 ± 60.5
Anesthetized	LVEDP (mmHg)	5.4 ± 4.7	8.6 ± 6.8
	dp/dt max (mmHg/s)	7119 ± 2109	5823 ± 2008 *
	dp/dt min (mmHg/s)	−6454 ± 1956	−5058 ± 1935 *
	Heart rate (bpm)	401 ± 57.4	417.2 ± 50.8

Stats are for all animals MI v Con, * *p* < 0.05.

**Table 2:** Hemodynamics and echocardiography parameters of sham and MI mice by sex.

		Sham		MI	
		Male	Female	Male	Female
	Number (Female)	19	7	22	7
	Body weight (g)	27 ± 1.5	19.6 ± 1.7 *	28 ± 1.7	20.1 ± 1.7 *
	Heart weight (mg)	152.4 ± 34.6	127.9 ± 11.9	213.4 ± 91.2 #	167.3 ± 38.0 †
	Lung weight (mg)	198.2 ± 39.8	97.9 ± 17.3 *	199.0 ± 63.8	156.4 ± 30.6 †
	HW/BW ratio	5.5 ± 1.1	6.5 ± 0.4 *	7.6 ± 3.5 #	8.2 ± 2.1
	LW/BW ratio	7.2 ± 1.5	5.1 ± 1.3 *	7.1 ± 2.1	7.6 ± 1.6 †
ECHO	Infarct (%)	-	-	38.4 ± 23.1	52.1 ± 14.2
	Ejection fraction (%)	57.5 ± 8.6	57.2 ± 9.9	25.4 ± 9.9 #	24.6 ± 14.2 †
	Fractional shortening (%)	15.9 ± 5.0	15.5 ± 8.8	8.2 ± 5.5 #	6.1 ± 4.2 †
	Cardiac output (mL/min)	15.5 ± 3.0	9.6 ± 2.5 *	12.1 ± 3.6 #	11.1 ± 6.2
	Heart rate (bpm)	468 ± 43.0	435.9 ± 68.9	489 ± 54.6	424.3 ± 57.4 *
Hemodynamics	LVEDP (mmHg)	4.6 ± 4.4	7.8 ± 5.0	7.6 ± 6.7	11.8 ± 6.5
	dp/dt max (mmHg/s)	7901 ± 1683	4770 ± 1419 *	6298 ± 2054 #	4329 ± 791 *
	dp/dt min (mmHg/s)	7235 ± 1517	4110 ± 1164 *	5572 ± 1939 #	3442 ± 542 *
	Heart rate (bpm)	423 ± 46.3	336 ± 33.3 *	431 ± 49.2	375 ± 29.1 †

All values are expressed as mean ± SD. * *p* < 0.05, male vs female within group. # *p* < 0.05, male sham to MI. † *p* < 0.05, female sham to MI.

**Table 3:** Hemodynamic and echo data of sham and MI rats with stellate GFP or Nrf2 overexpression.

		Sham		MI	
		GFP	GFP-Nrf2	GFP	GFP-Nrf2
		(n = 10)	(n = 9)	(n = 11)	(n = 12)
	Body (g)	348.4 ± 24.2	394.9 ± 83.3	360.3 ± 23.0	390.7 ± 53.2
	Heart (mg)	1105 ± 173	1218 ± 242	1419 ± 241 *	1508 ± 231 *
	Lung (mg)	1418 ± 104	1502 ± 202	2371 ± 924 *	2513 ± 1411 *
	HW/BW	3.2 ± 0.4	3.1 ± 0.3	3.9 ± 0.7 *	3.9 ± 0.6 *
	LW/BW	4.1 ± 0.2	3.9 ± 0.7	6.6 ± 2.7 *	6.5 ± 3.8 *
	Infarct (%)	-	-	30.1 ± 15.3	33.5 ± 16.2
ECHO	Ejection fraction (%)	74.2 ± 4.9	74.9 ± 3.1	35.6 ± 10.7 *	34.7 ± 10.3 *
	Fractional shortening (%)	44.9 ± 4.5	45.4 ± 3.0	18.4 ± 6.6 *	17.8 ± 5.9 *
	Cardiac output (mL/min)	89.4 ± 18.9	94.1 ± 31.1	78.1 ± 18.9	85.3 ± 13.6
	Heart rate (bpm)	325 ± 38	323 ± 44	318 ± 32	339.4 ± 57.7
Hemodynamics	LVEDP (mmHg)	11.3 ± 4.5	8.8 ± 3.9	22.5 ± 11.7 *	21.8 ± 9.5 *
	dp/dt max (mmHg/s)	6936 ± 718	8139 ± 1462 #	5184 ± 1307 *	5525 ± 979 *
	dp/dt min (mmHg/s)	−6884 ± 1127	−7611 ± 2014	−4150 ± 1145 *	−4593 ± 1040 *
	Heart rate (bpm)	255 ± 65	308 ± 37 #	288 ± 27	277 ± 70.2
	Blood pressure (mmHg)	110.3 ± 79.3	96.4 ± 13.5	85.0 ± 15.7	95.8 ± 54.2

* *p* < 0.05, MI to sham—for both GFP and GFP Nrf-2. # *p* < 0.05, Sham-GFP to Sham-Nrf2.

## Data Availability

All original data are available from the authors upon reasonable request.
